# Exploring Entanglement Spectra and Phase Diagrams in Multi-Electron Quantum Dot Chains

**DOI:** 10.3390/e27050479

**Published:** 2025-04-29

**Authors:** Guanjie He, Xin Wang

**Affiliations:** 1 Department of Physics, City University of Hong Kong, Tat Chee Avenue, Kowloon, Hong Kong SAR, China; guanjiehe2-c@my.cityu.edu.hk; 2 Shenzhen Research Institute, City University of Hong Kong, Shenzhen 518057, China; 3 Quantum Science Center of Guangdong-Hong Kong-Macao Greater Bay Area, Shenzhen 518045, China

**Keywords:** Hubbard model, entanglement, quantum dot

## Abstract

We investigate the entanglement properties in semiconductor quantum dot systems modeled by the extended Hubbard model, focusing on the impacts of potential energy variations and electron interactions within a four-site quantum dot spin chain. Our study explores local and pairwise entanglement across configurations with electron counts N=4 and N=6, under different potential energy settings. By adjusting the potential energy in specific dots and examining the entanglement across various interaction regimes, we identify significant variations in the ground states of quantum dots. We extend this analysis to larger systems with L=6 and L=8, comparing electron counts N=L and N=L+2, revealing sharper entanglement transitions and reduced finite-size effects as the system size increases. Our results show that local potential shifts and the Coulomb interaction strength lead to notable redistributions of the electron configurations in the quantum dot spin chain, significantly affecting the entanglement properties. These changes are depicted in phase diagrams that highlight entanglements’ dependencies on the interaction strengths and potential energy adjustments, illustrating complex entanglement dynamics shifts triggered by interdot interactions.

## 1. Introduction

Quantum entanglement plays a crucial role in various fields of quantum physics, including quantum communication and quantum information processing [1,2]. In condensed matter physics, especially in many-body quantum systems, quantum entanglement serves as a fundamental criterion for quantum phase transitions and many-body localization [3,4,5,6,7]. Among various systems, semiconductor quantum dots have emerged as scalable, implementable, and precisely controllable [8,9,10,11,12,13,14,15,16] platforms for the simulation of many-body systems of interest, particularly the Fermi–Hubbard physics [17,18,19,20,21,22,23]. The Fermi–Hubbard model provides a common framework describing quantum dot systems in the regime of low temperatures and strong Coulomb interactions, finding extensive application in the physical realization of quantum information processing and entanglement creation [24,25,26,27,28,29,30,31,32]. Consequently, a comprehensive understanding of quantum dots from the perspective of Fermi–Hubbard physics becomes imperative.

High-fidelity qubit gate operations [33,34] and noise suppression schemes [35] commonly applied to conventional quantum dot systems, where each dot accommodates at most two electrons, traditionally rely on the monotonically increasing behavior of exchange energy as a function of detuning [36,37,38,39,40]. However, recent investigations [41,42,43,44,45,46,47,48,49,50,51,52] have revealed the interesting properties of specific quantum dots capable of hosting more than two electrons, such as the non-monotonic behavior of exchange energy with distinct sweet spots [50,53], fast spin exchange dynamics [54], superexchange interactions between non-neighboring dots [55,56,57], and resilience to noise [53,58,59,60]. These properties can be attributed to the influence of higher excited orbitals and can be effectively understood within the framework of the full configuration interaction [61] and the extended Hubbard model (EHM), which incorporates multiple energy levels.

The entanglement spectrum of the one-dimensional EHM in its ground state is well understood [62,63,64,65]. Consequently, in the case of a half-filled system, the entanglement properties of a quantum dot spin chain can be effectively explained [66]. However, when there is a tilted potential energy difference among the dots, the mirror symmetry of the system is broken, which leads to tunable entanglement values through the application of precise electron control using external electric fields [67]. These previous works motivated us to investigate the entanglement spectrum of a quantum dot spin chain where each dot incorporates multiple energy levels, which could be measured indirectly in the future in multiple ways [68,69,70,71].

In this study, we investigate the entanglement patterns of the ground states of multi-electron quantum dot systems using the EHM, which incorporates multiple orbitals within each dot. Our specific focus lies in characterizing the entanglement properties of one-site and two-site reduced density matrices. By computing and analyzing the entanglement spectrum for various system sizes, we uncover notable findings. Firstly, when there are no potential energy differences among the dots, the multi-electron quantum dot system can be accurately described by the EHM, either in a half-filled state or a non-half-filled state, depending on the total electron number. However, when a selected dot within the chain exhibits a potential energy difference relative to its neighboring dots, distinct system phases and phase boundaries emerge in the entanglement spectrum. These phases depend on the coupling strengths and potential energy difference values. The emergence of these phases indicates that the presence of a selected dot with a potential energy ladder profoundly impacts the electron configuration in its vicinity. This influence is more pronounced in small systems and limited in larger-sized systems due to the size effect.

This paper is organized as follows. In Section 2, we present the EHM as a suitable framework to describe multi-electron quantum dot chain systems. Section 3 introduces the definition of one-site and two-site reduced density matrices and entanglement entropy for these systems. Our main results are presented in Section 4, starting with an examination of a system size of L=4 and electron numbers N=4 and N=6. We analyze the entanglement spectrum properties with and without potential energy differences. Furthermore, we extend our analysis to larger system sizes, specifically L=6 and L=8, with electron numbers N=L and N=L+2, and compare these to the L=4 case, while also considering the behavior as *L* approaches infinity. Finally, we summarize our findings and provide concluding remarks in Section 5.

## 2. Extended Hubbard Model

**Figure 1:**
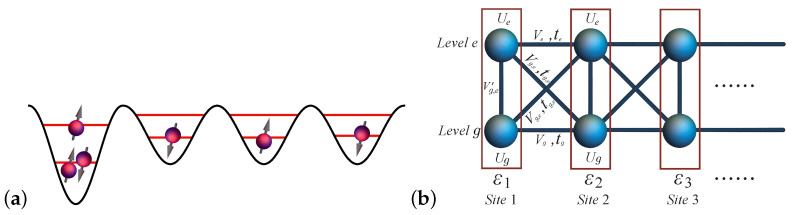
(**a**) Schematic illustration of a L=4 multi-electron quantum dot spin chain system hosting N=6 electrons. (**b**) In the two-level case, the equivalent asymmetric Hubbard ladder is described by a Hamiltonian (1). The box indicates, for each site *i*, that electrons at different energy levels have the same detuning energy $\varepsilon_{i}$. *g* indicates the ground level and *e* the first excited level.

We consider a multiple-quantum-dot system (MQD) (schematically shown in Figure 1), described by an EHM with short-range Coulomb interactions and tunneling restricted to nearest-neighbor sites within the same energy level and the nearest-neighbor energy level. The model can be described by the following Hamiltonian:

(1) $$\begin{array}{cc} H= & -\sum_{i,\nu ,\sigma }\left(t_{\nu }c_{i,\nu ,\sigma }^{†}c_{i+1,\nu ,\sigma }+H.c.\right)-\sum_{i,\nu ,\bar{\nu },\sigma }\left(t_{\nu ,\bar{\nu }}c_{i,\nu ,\sigma }^{†}c_{i+1,\bar{\nu },\sigma }+H.c.\right)\\ \; & +\sum_{i,\nu ,\sigma }V_{\nu }n_{i,\nu ,\sigma }n_{i+1,\nu ,\sigma^{\prime }}+\sum_{i,\nu ,\bar{\nu },\sigma }\left(V_{\nu ,\bar{\nu }}n_{i,\nu ,\sigma }n_{i+1,\bar{\nu },\sigma^{\prime }}+V_{\nu ,\bar{\nu }}^{\prime }n_{i,\nu ,\sigma }n_{i,\bar{\nu },\sigma^{\prime }}\right)+\sum_{i,\nu }U_{\nu }n_{i,\nu ↓}n_{i,\nu ↑}\\ \; & +\sum_{i,\sigma }\varepsilon_{i,\sigma }n_{i\sigma }, \end{array}$$

where *i* indicates the quantum dot site; ν and $\bar{\nu }$ denote different orbital levels, which can be either ground orbitals (*g*) or excited orbitals (*e*); and σ and $\sigma^{\prime }$ refer to the spins that are either up or down. $\varepsilon_{i,\sigma }$ is the potential energy at dot *i*; note that, although, in one quantum dot, electrons can occupy different orbitals, they share the same potential energy. $t_{\nu }$ is the tunneling energy between the *i*th and (i+1)th sites at the νth orbital level. $t_{\nu ,\bar{\nu }}$ is the tunneling energy between the *i*th site at the νth orbital level and the (i+1)th site at the $\bar{\nu }$th orbital level, i.e., $t_{g,e}$ or $t_{e,g}$. $U_{\nu }$ denotes the on-site Coulomb interaction at the νth orbital level. $V_{\nu }$ is the nearest direct Coulomb interaction between the *i*th and (i+1)th sites at the νth orbital. $V_{\nu ,\bar{\nu }}$ is the nearest direct Coulomb interaction between the *i*th site at the νth orbital and the (i+1)th site at the $\bar{\nu }$th orbital, and, finally, $V_{\nu ,\bar{\nu }}^{\prime }$ is the nearest direct Coulomb interaction between the νth orbital and $\bar{\nu }$th orbital at the *i*th site, i.e., $V_{g,e}$, $V_{e,g}$, $V_{g,e}^{\prime }$, $V_{e,g}^{\prime }$.

According to the Pauli exclusion principle, electrons have four occupation states ${|v\rangle }_{i,\nu }$=${|0\rangle }_{i,\nu }$, ${|↑\rangle }_{i,\nu }$, ${|↓\rangle }_{i,\nu }$, ${|↑↓\rangle }_{i,\nu }$ in the νth orbital of the *i*th site. Thus, the dimension of the Fock space for an *L*-site MQD chain with *K* orbitals per site is $4^{LK}$. The configuration basis states are $|v_{1},v_{2},...,v_{L}\rangle$ = $\prod_{i=1}^{L}{|v_{i}\rangle }_{i}$, where $|v_{i}\rangle_{i}$ = $\prod_{\nu =1}^{K}{|v\rangle }_{i,\nu }$ represents the configuration basis for the i-th site. In this work, we numerically study MQD chains with *N* and N+2 electrons in L=N sites and total spin S=0 systems, restricting our analysis to the ground and first excited orbital states (ν=g,e) for each quantum dot. All S=0 electron configurations are considered for the L=4 and L=6 systems, while, for L=8, only energetically favorable configurations are included.

## 3. Reduced Density Matrices and Entanglement

We first obtain the ground state (GS) $|\psi_{\mathrm{GS}}\rangle$ of the system by diagonalizing the effective Hamiltonian. All configurations are considered for L = 4 and L = 6 systems, while, for L = 8, only energetic favorable configurations are considered due to the Hilbert space size. The GS can be expressed as the linear superposition of all possible electron configuration basis states $|\psi_{m}\rangle$ in the occupation number representation $|v_{1},v_{2}...v_{L}\rangle$:

(2) $$|\psi_{\mathrm{GS}}\rangle =\sum_{m}c_{m}|\psi_{m}\rangle ,$$

where $c_{m}$ denotes the coefficients of the superposition.

The density matrix $\rho_{\mathrm{GS}}$ of the entire system can be expressed as the sum of the occupation probabilities $P_{m}$ of all electron configurations $|\psi_{m}\rangle$:

(3) $$\rho_{\mathrm{GS}}=\sum_{m}P_{m}|\psi_{m}\rangle \langle \psi_{m}|.$$

To analyze entanglement, we divide the full system into subsystems A and B. The reduced density matrix $\rho_{A}$ for subsystem A is obtained by taking the partial trace of $\rho_{\mathrm{GS}}$:

(4) $$\rho_{A}={\mathrm{Tr}}_{B}\rho_{\mathrm{GS}}.$$

The von Neumann entropy $E(\rho_{A})$ measures the entanglement between subsystem A and the remaining subsystem B and is defined as

(5) $$E\left(\rho_{A}\right)=-\mathrm{Tr}\left(\rho_{A}\log_{2}\rho_{A}\right).$$

### 3.1. Local Entanglement of Multi-Electron Quantum Dot

In the two orbitals, and within the parameters considered, electrons prefer to doubly occupy ground states before filling the first excited states. Therefore, the state space of a single site is spanned by nine bases: ${\{|0,0\rangle ,|↑}_{g}$,${0\rangle ,|↓}_{g}{,0\rangle ,|↑}_{g}{↓}_{g}{,0\rangle ,|↑}_{g}{,↓}_{e}{\rangle ,|↓}_{g}{,↑}_{e}{\rangle ,|↑}_{g}{↓}_{g}{,↑}_{e}{\rangle ,|↑}_{g}{↓}_{g}{,↓}_{e}{\rangle ,|↑}_{g}{↓}_{g}$, ${↑}_{e}{↓}_{e}\rangle \}$. Here, $0_{g}$ and $0_{e}$ represent cases with no electron occupying the ground and the first excited orbital, respectively. ${↑}_{g}$, ${↓}_{g}$, ${↑}_{e}$, ${↓}_{e}$ stand for an electron with spin up or down, indicated as the arrow staying in the ground (g) and the first excited orbital (e) indicated in the subscript, respectively.

The two-level one-site reduced density matrix for site *i* can be written as

(6) $$\rho_{i}={\mathrm{Tr}}_{L\setminus \left\{i\right\}}\left(\rho_{\mathrm{GS}}\right),$$

with ${\mathrm{Tr}}_{L\setminus \left\{i\right\}}$ as the partial trace over all sites except site *i*. Expressed in terms of the basis ${\{|0,0\rangle ,|↑}_{g}$,${0\rangle ,|↓}_{g}{,0\rangle ,|↑}_{g}{↓}_{g}{,0\rangle ,|↑}_{g}{,↓}_{e}{\rangle ,|↓}_{g}{,↑}_{e}{\rangle ,|↑}_{g}{↓}_{g}{,↑}_{e}{\rangle ,|↑}_{g}{↓}_{g}{,↓}_{e}{\rangle ,|↑}_{g}{↓}_{g}$, ${↑}_{e}{↓}_{e}\rangle \}$, $\rho_{i}$ can be written as a 9×9 matrix as follows:

$\rho_{i}$=$\left(\begin{array}{ccccccccc} v_{i,1} & & & & & & & & \\ & v_{i,2} & & & & & & & \\ & & v_{i,3} & & & & & & \\ & & & v_{i,4} & v_{i,a} & v_{i,b} & & & \\ & & & v_{i,a} & v_{i,5} & v_{i,c} & & & \\ & & & v_{i,b} & v_{i,c} & v_{i,6} & & & \\ & & & & & & v_{i,7} & & \\ & & & & & & & v_{i,8} & \\ & & & & & & & & v_{i,9} \end{array}\right)$. Here, $v_{i,m}$(m=1,2,...,9), $v_{i,a}$, $v_{i,b}$ and $v_{i,c}$ are determined by the potential energy ε of different dots and quantity *U*. In the half-filled case, when there is no potential energy difference among all quantum dots, the local reduced density matrix $\rho_{i}$ can be simplified to a one-energy-level case [65], with

(7a) $$\begin{array}{c} v_{i,1}=1-v_{i,4}+v_{i,2}+v_{i,3}, \end{array}$$

(7b) $$\begin{array}{c} v_{i,2}=\langle n_{i,g,↑}\rangle -v_{i,4}, \end{array}$$

(7c) $$\begin{array}{c} v_{i,3}=\langle n_{i,g,↓}\rangle -v_{i,4}, \end{array}$$

(7d) $$\begin{array}{c} v_{i,4}=\mathrm{Tr}\left(n_{i,g,↑}n_{i,g,↓}\rho_{i}\right)=\langle n_{g↑}n_{g↓}\rangle , \end{array}$$

(7e) $$\begin{array}{c} v_{i,a}=v_{i,b}=v_{i,c}=0, \end{array}$$

(7f) $$\begin{array}{c} v_{i,5}=v_{i,6}=v_{i,7}=v_{i,8}=v_{i,9}=0. \end{array}$$

When there is a potential energy difference between quantum dots—specifically, in our case, where only one site’s potential energy is modified while the others remain unchanged—the contributions from $v_{i,5}$, $v_{i,6}$, $v_{i,7}$, $v_{i,8}$, $v_{i,9}$, $v_{i,a}$, $v_{i,b}$, and $v_{i,c}$ must be taken into account. Therefore, the above expression of $v_{i,m}$ does not hold. However, we can still derive that $v_{i,2}=v_{i,3}$, $v_{i,5}=v_{i,6}$, and $v_{i,7}=v_{i,8}$. In particular, within the parameters that we set (which will be explained in detail later), the bases of ${|↑}_{g},{↓}_{e}\rangle$ and ${|↓}_{g},{↑}_{e}\rangle$ are energetically unfavorable and therefore have no contribution, leading to $v_{i,5},v_{i,6},v_{i,a},v_{i,b},v_{i,c}∼0$ at any potential $V_{i}$. Thus, $\rho_{i}$ can be represented as a 7×7 diagonal matrix:

(8) $$\begin{array}{cc} \rho_{i}= & v_{i,1}|0_{g},0_{e}\rangle \langle 0_{g},0_{e}|+v_{i,2}{|↑}_{g},0_{e}{\rangle \langle ↑}_{g},0_{e}|\\ \; & +v_{i,3}{|↓}_{g},0_{e}{\rangle \langle ↓}_{g},0_{e}|+v_{i,4}{|↑}_{g}{↓}_{g},0_{e}{\rangle \langle ↑}_{g}{↓}_{g},0_{e}|\\ \; & +v_{i,7}{|↑}_{g}{↓}_{g}{,↑}_{e}{\rangle \langle ↑}_{g}{↓}_{g}{,↑}_{e}|+v_{i,8}{|↑}_{g}{↓}_{g}{,↓}_{e}{\rangle \langle ↑}_{g}{↓}_{g},{↓}_{e}|\\ \; & +v_{i,9}{|↑}_{g}{↓}_{g}{,↑}_{e}{↓}_{e}{\rangle \langle ↑}_{g}{↓}_{g}{,↑}_{e}{↓}_{e}|. \end{array}$$

**Figure 2:**
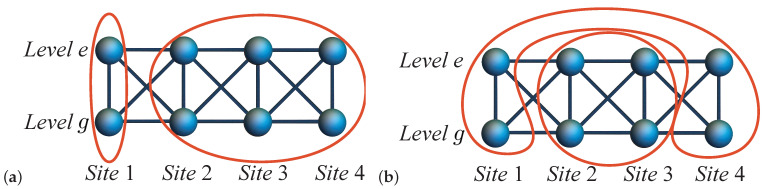
Illustration of bipartite entanglement and quantum states in L=4 two-level system. (**a**) Local entanglement $E(\rho_{1})$ and (**b**) pairwise entanglement $E\left(\rho_{14}\right)=E\left(\rho_{23}\right)$. The red circle indicates the selected partition. *g* indicates the ground level and *e* the first excited level.

For the N=4 system, there are four distinct approaches to analyzing local bipartite entanglement: $E(\rho_{1})$, $E(\rho_{2})$, $E(\rho_{3})$, and $E(\rho_{4})$. An example of this can be seen in Figure 2a, which shows the local entanglement $E(\rho_{1})$.

### 3.2. Pairwise Entanglement of Multi-Electron Quantum Dot

Similarly, for site *i* and site *j*, the two-site reduced density matrix can be written as

(9) $$\rho_{ij}={\mathrm{Tr}}_{L\setminus \left\{i,j\right\}}\left(\rho_{\mathrm{GS}}\right),$$

with ${\mathrm{Tr}}_{L\setminus \left\{i,j\right\}}$ as the partial trace over all sites except sites *i* and *j*. As depicted in Figure 2b, according to the nine bases considered for a single site in Section 3.1, the electrons in two sites with two orbitals have $9^{2}=81$ possible configurations. With respect to these bases, $\rho_{ij}$ can be described as an 81×81 matrix. Similarly to the one-site case, where we dropped two energetically unfavorable bases ${|↑}_{g},{↓}_{e}\rangle$ and ${|↓}_{g},{↑}_{e}\rangle$, $\rho_{ij}$ can be described as a 49×49 matrix since electrons in two sites have $7^{2}=49$ occupation probabilities. There are three possible approaches to analyzing pairwise bipartite entanglement for the N=4 system: $E(\rho_{12})$ and $E(\rho_{34})$, $E(\rho_{13})$ and $E(\rho_{24})$, $E(\rho_{14})$ and $E(\rho_{23})$. Figure 2b demonstrates one possible bipartite pairwise entanglement, $E(\rho_{14})$ and $E(\rho_{23})$.

## 4. Results

In our quantum dot system setup, we have defined a set of parameters that can represent the properties of multi-electron dots [49,50]. Accordingly, we set the tunneling energy between the nearest sites to be larger for lower orbitals and smaller for higher orbitals. This means that the tunneling between two ground orbitals is the greatest, followed by the tunneling between one ground orbital and one excited orbital and, finally, the tunneling between two excited orbitals, i.e., $t_{e}<t_{g,e}<t_{g}$. Similarly, within one single dot or between two nearest dots, the on-site Coulomb interaction energy and the nearest direct Coulomb interaction energy from higher orbitals are larger than those from lower energy levels, since an electron that occupies a higher orbital requires more energy, i.e., $U_{g}<V_{g,e}^{\prime }<U_{e}$ and $V_{g}<V_{g,e}<V_{e}$. The numerical relation between $V_{\nu }$ and $U_{\nu }$ is referenced from [13,25,50,72,73], satisfying a strong repulsive on-site interaction regime in the EHM [65], i.e., $V_{\nu }<U_{\nu }$ and $V_{g,e}<V_{g,e}^{\prime }$. In a one-dimensional EHM at half-filling, the ratio between on-site Coulomb interaction $U_{\nu }$ and the nearest direct Coulomb interaction energy $V_{\nu }$ will lead to a charge density wave (CDW) and spin density wave (SDW) in the strong coupling limit regime [65]. Specifically, for $U_{g}>2V_{g}$, the ground state is a staggered charge density wave, and, for $U_{g}<2V_{g}$, the ground state is a staggered spin density wave. These spin order properties will also be apparent in our simulation results due to the chosen parameters; therefore, our discussion will be split into two parts: $U_{g}>2V_{g}$ and $U_{g}<2V_{g}$. In this study, we have set our parameters as follows: $V_{g}=\alpha U_{g}$, $V_{g,e}=\alpha V_{g,e}^{\prime }$, $Ve=\alpha U_{e}$, $V_{g,e}^{\prime }=1.5U_{g}$, $U_{e}=2U_{g}$, $t_{e}=0.3t_{g}$, $t_{g,e}=0.6t_{g}$ [41,49,74]. Here, according to the literature, the coupling strength ratio of α can be either set as 0.2 [72] or 0.7 [13,25,73], and $U=U_{g}/t_{g}$ is the main quantity parameter in the results.

### 4.1. Local Entanglement at $\varepsilon_{1}=\varepsilon_{2}=\varepsilon_{3}=\varepsilon_{4}=0$

**Figure 3:**
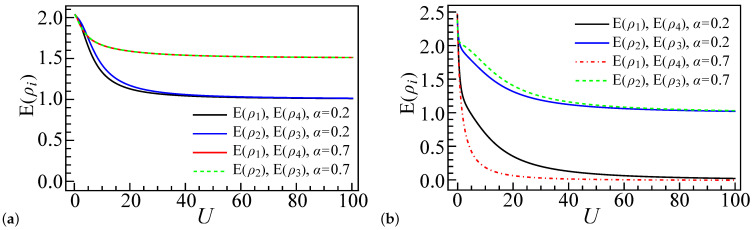
Local entanglement $E(\rho_{i})$ profiles for a four-site quantum dot system (L=4) with coupling strengths α=0.2 or α=0.7, displayed as a function of interaction strength *U*. Panels (**a**,**b**) correspond to systems with four (N=4) and six (N=6) electrons, respectively, with zero detuning energy ($\varepsilon_{i}=0$) at all sites. The entanglement measures $E(\rho_{1})$ and $E(\rho_{4})$ are equivalent, as are $E(\rho_{2})$ and $E(\rho_{3})$.

**Figure 4:**
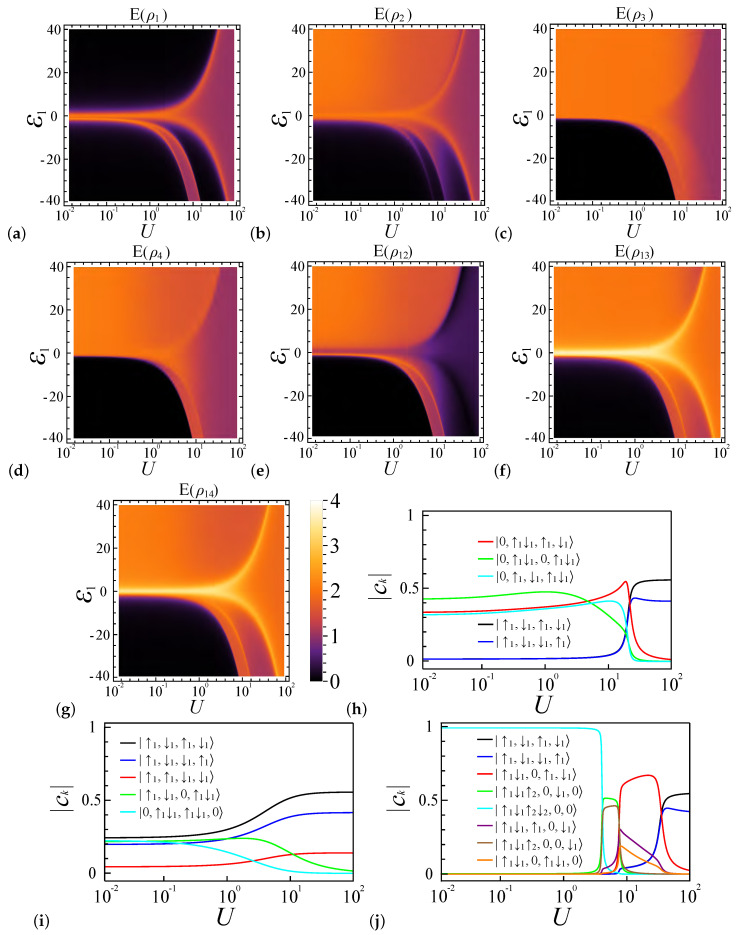
Entanglement phase diagrams for a quantum dot system with four sites (L=4) and four electrons (N=4) under a coupling strength ratio of α=0.2. These diagrams are plotted as functions of the interaction strength *U* and the potential energy $\varepsilon_{1}$. (**a**–**d**) Local entanglement measures $E(\rho_{1})$, $E(\rho_{2})$, $E(\rho_{3})$, and $E(\rho_{4})$, respectively. (**e**–**g**) Pairwise entanglement for dot pairs $E(\rho_{12})$, $E(\rho_{13})$, and $E(\rho_{14})$. (**h**–**j**) illustrate the proportions of selected advantageous electron configurations within the system’s ground state, highlighting the influence of interaction parameters on system behavior; they represent cases where $\varepsilon_{1}=20$, $\varepsilon_{1}=0$, and $\varepsilon_{1}=-20$, respectively.

Starting with an analysis of the local entanglement in the smallest system size (L=4) for both electron number scenarios (N=4 and N=6), we first consider the case of N=4 with α=0.2, as depicted in Figure 3a. It is apparent that, for N=4, the local entanglement at the end sites ($E\left(\rho_{1}\right)=E\left(\rho_{L}\right)$) is both equal to and less than the local entanglement of the inner sites. This phenomenon arises from the preference of the end sites for single occupancy over the middle sites, particularly as the repulsive interaction increases [66]. With the increase in the repulsive interaction *U* in the four-dot-four-electron system, specific configurations, such as ${|↑}_{g}{,↓}_{g}{,↑}_{g},{↓}_{g}\rangle$, ${|↓}_{g}{,↑}_{g}{,↓}_{g},{↑}_{g}\rangle$, ${|↑}_{g}{,↑}_{g}{,↓}_{g},{↓}_{g}\rangle$, ${|↓}_{g}{,↓}_{g}{,↑}_{g},{↓}_{g}\rangle$, ${|↑}_{g}{,↓}_{g}{,↓}_{g},{↑}_{g}\rangle$, and ${|↓}_{g}{,↑}_{g}{,↑}_{g},{↓}_{g}\rangle$, progressively dominate the ground state, as illustrated in Figure 4i.

**Figure 5:**
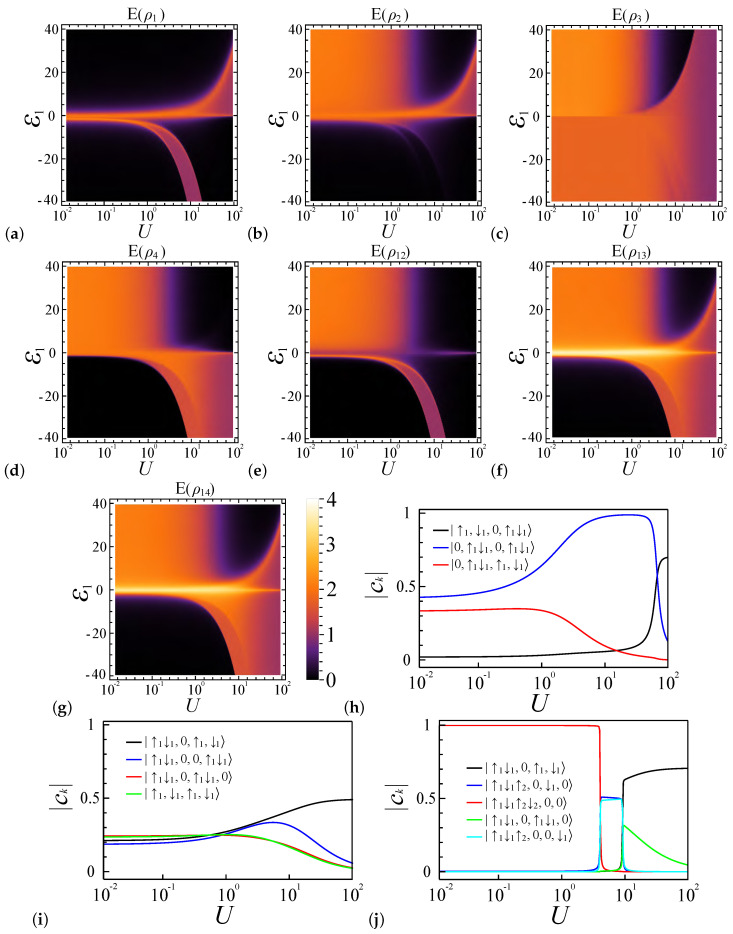
Entanglement characteristics of a four-dot (L=4), four-electron (N=4) quantum dot system at a coupling ratio of α=0.7. Diagrams are plotted against interaction strength *U* and potential energy $\varepsilon_{1}$. (**a**–**d**) Local entanglement measures $E(\rho_{1})$ to $E(\rho_{4})$. (**e**–**g**) Pairwise entanglement for dot pairs $E(\rho_{12})$, $E(\rho_{13})$, and $E(\rho_{14})$. The dominant electron configurations in the ground state corresponding to $\varepsilon_{1}=20$, $\varepsilon_{1}=0$, and $\varepsilon_{1}=-20$ are represented by (**h**–**j**), respectively.

For N=4 and α=0.7, with $U_{g}<2V_{g}$, akin to the behavior observed in the charge density wave in large chain systems [65], electrons in a single dot tend to favor double occupancy over single occupancy. In a four-dot system, as *U* increases, specific electron configurations such as ${|↑}_{g}{,↓}_{g}{,0,↑}_{g}{↓}_{g}\rangle$, ${|↓}_{g}{,↑}_{g}{,0,↑}_{g}{↓}_{g}\rangle$, ${|↑}_{g}{↓}_{g}{,0,↑}_{g},{↓}_{g}\rangle$, and ${|↑}_{g}{↓}_{g}{,0,↓}_{g},{↑}_{g}\rangle$ come to dominate the ground state configuration, as depicted in Figure 5i (the above four states are all represented by ${|↑}_{g}{↓}_{g}{,0,↑}_{g},{↓}_{g}\rangle$ since they can be equally treated). This is related to the small size effect, since, in such a system, these configurations are the most energetically favorable. Moreover, in Figure 3a, it is evident that $E\left(\rho_{1}\right)=E\left(\rho_{4}\right)$ and $E\left(\rho_{2}\right)=E\left(\rho_{3}\right)$, as all sites have an equal ratio of the four configurations of |0〉, ${|↑}_{g}\rangle$, ${|↓}_{g}\rangle$, and ${|↑}_{g}{↓}_{g}\rangle$. Specifically, $E(\rho_{1})$ is almost equal to $E(\rho_{2})$, with any differences being brought about by configuration states such as ${|↑}_{g}{↓}_{g}{,0,0,↑}_{g}{↓}_{g}\rangle$, as illustrated in Figure 5h.

**Figure 6:**
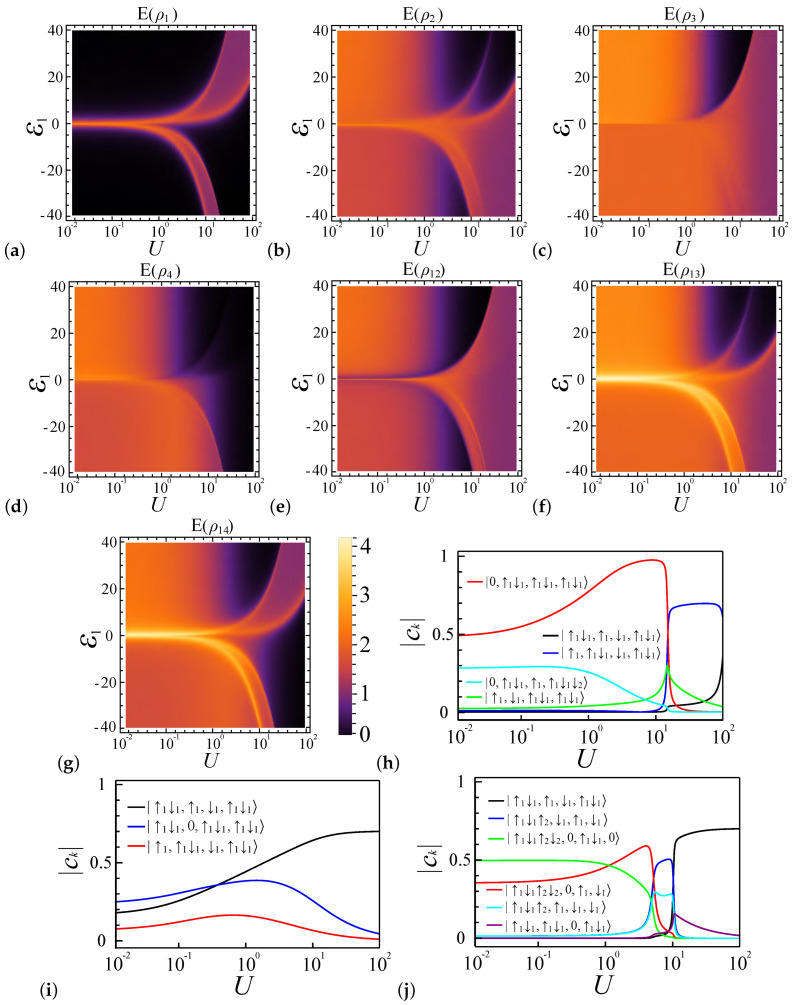
Entanglement profiles for a four-site (L=4), six-electron (N=6) quantum dot system with a coupling strength of α=0.2. Charts are graphed according to interaction strength *U* and potential energy $\varepsilon_{1}$. (**a**–**d**) depict local entanglement levels $E(\rho_{1})$ through $E(\rho_{4})$. (**e**–**g**) depict pairwise entanglement between dot pairs $E(\rho_{12})$, $E(\rho_{13})$, and $E(\rho_{14})$. (**h**–**j**) show the predominant electron configurations in the system’s ground state, corresponding to scenarios where $\varepsilon_{1}=20$, $\varepsilon_{1}=0$, and $\varepsilon_{1}=-20$, respectively.

In the L=4, N=6, and α=0.2 system, the entanglement is as shown in Figure 3b. Due to the presence of two extra electrons (compared to the N=4 case), the electron configurations of ${|↑}_{g}{↓}_{g}{,↑}_{g}{,↓}_{g}{,↑}_{g}{↓}_{g}\rangle$, ${|↑}_{g}{↓}_{g}{,↑}_{g}{,↑}_{g}{↓}_{g},{↓}_{g}\rangle$, and ${|↑}_{g}{↓}_{g}{,↑}_{g}{↓}_{g}{,↑}_{g},{↓}_{g}\rangle$ have the primary contributions to the system ground state $\psi_{\mathrm{GS}}$, as shown in Figure 6i. To facilitate the later discussion, we also introduce a notation describing the number of electrons in different sites. For example, |••,•,•,••〉, |••,•,••,•〉, and |••,••,•,•〉 represent the three aforementioned states’ occupancy, respectively, where • or •• represents a site occupied by one electron or two electrons, respectively. We also use ◦ to express an empty site, so |••,◦,••,••〉 represents a case where site 1, site 3, and site 4 are doubly occupied, while site 2 has no electron.

In the weak coupling regime, where U∼0, all electron configuration components have roughly the same proportions; thus, $E(\rho_{i})$ at U∼0 have similar values. As *U* increases, the local entanglement of the end dots decreases more rapidly than that of the inner dots from the middle of the chain, and this rate of descent is even faster in the N=6 case than the N=4 case with α=0.2. This is due to the increasing dominance of the |••,•,•,••〉 configuration in the ground state, as depicted in Figure 6i. At U≫1, the inner dots tend to favor single occupancy, thereby resulting in similar values for $E(\rho_{2})$ and $E(\rho_{3})$ for both N=4 and N=6, while the end dots in the N=6 case favor double occupancy, leading to a rapid decrease in the entanglement value.

**Figure 7:**
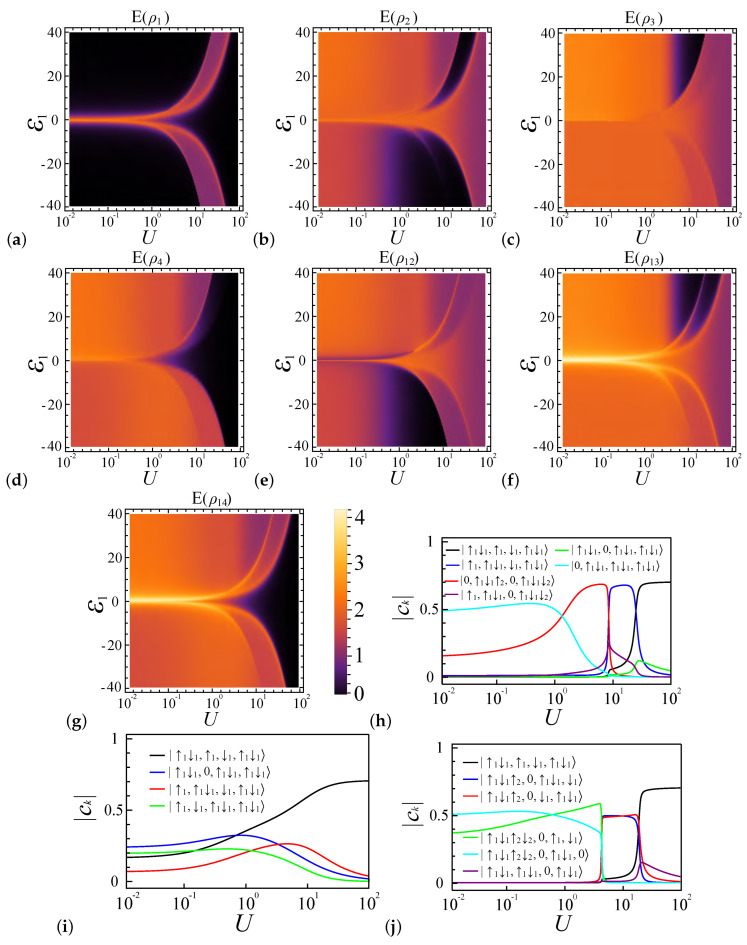
Entanglement profiles for a four-site (L=4), six-electron (N=6) quantum dot system with a coupling strength of α=0.7. Charts are graphed according to interaction strength *U* and potential energy $\varepsilon_{1}$. (**a**–**d**) depict local entanglement levels $E(\rho_{1})$ through $E(\rho_{4})$. (**e**–**g**) depict pairwise entanglement between dot pairs $E(\rho_{12})$, $E(\rho_{13})$, and $E(\rho_{14})$. (**h**–**j**) illustrate the dominant electron configurations in the system’s ground state for $\varepsilon_{1}=20$, $\varepsilon_{1}=0$, and $\varepsilon_{1}=-20$, respectively.

For N=6 and α=0.7, the system tends to favor double occupancy. Hence, the configurations |••,•,•,••〉, |••,◦,••,••〉 (also |••,••,◦,••〉) have a greater presence in the ground state compared to the α=0.2 case, as illustrated in Figure 7i. When compared to Figure 6i, the maximal probability of |••,◦,••,••〉 and |••,••,◦,••〉 in Figure 7i has shifted toward a smaller *U*. This suggests that, in the α=0.7 configuration, sites 1 and 4 favor double occupancy more than in the α=0.2 case, resulting in a steeper decline in $E(\rho_{1})$ and $E(\rho_{4})$; meanwhile, for sites 2 and 3, $E(\rho_{2})$ and $E(\rho_{3})$ decrease more slowly in the α=0.7 scenario compared to α=0.2, as the configuration |•,••,•,••〉 (Figure 7i) is the second-largest contributor to the behavior of sites 2 and 3 for α=0.7, whereas, in α=0.2, the configuration |••,◦,••,••〉 (Figure 6i) plays this role.

### 4.2. Pairwise Entanglement at $\varepsilon_{1}=\varepsilon_{2}=\varepsilon_{3}=\varepsilon_{4}=0$

**Figure 8:**
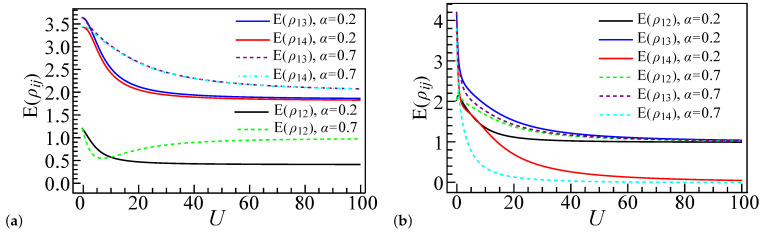
These figures illustrate the pairwise entanglement metrics $E(\rho_{ij})$ for a four-site (L=4) quantum dot array, analyzed under two coupling strength scenarios, α=0.2 and α=0.7. Displayed as functions of the interaction parameter *U*, panel (**a**) details configurations with four electrons (N=4) and panel (**b**) with six electrons (N=6), all with zero detuning energy at each site ($\varepsilon_{i}=0$). The figures demonstrate equivalent entanglement values between dot pairs—specifically, $E(\rho_{12})$ with $E(\rho_{34})$, $E(\rho_{13})$ with $E(\rho_{24})$, and $E(\rho_{23})$ with $E(\rho_{14})$.

In the L=4 system, with all quantum dots having equal potential energy ($\varepsilon_{1}=\varepsilon_{2}=\varepsilon_{3}=\varepsilon_{4}=0$), mirror reflection symmetry ensures that the pairs of two-site reduced density matrices satisfy the relations $\rho_{12}=\rho_{34}$ and $\rho_{13}=\rho_{24}$. Additionally, due to the finite size effect inherent in the small system, it is observed that $\rho_{14}=\rho_{23}$, as illustrated in Figure 8.

For N=4 and α=0.2, the entanglement results of $E(\rho_{12})$, $E(\rho_{13})$, and $E(\rho_{14})$ align well with the theoretical predictions for non-interacting systems (α=0), as elucidated in Ref. [66] and depicted in Figure 8a. In the limit where U∼0, $E(\rho_{ij})$ has the same value for different α values since all Coulomb interactions are zero. Conversely, at α=0.7 with a positive *U* value, the system demonstrates a preference for electron configurations such as |••,◦,•,•〉 and |•,•,◦,••〉. This preference equilibrates the entanglement levels $E(\rho_{13})$ and $E(\rho_{14})$ within the strong coupling regime, as illustrated in Figure 8a. Within this regime, the probabilities for zero- and single-electron occupancy at sites 2 and 3 become comparable, as do the probabilities for single- and double-electron occupancy at sites 1 and 4, a phenomenon detailed in Figure 5i. Concerning $E(\rho_{12})$, as depicted in the same figure, the diminished favorability of the state |••,◦〉 for the first and second sites leads to a reduction in the prevalence of the state |••,◦,◦,••〉 as *U* increases. This reduction also leads to an increase in $E(\rho_{12})$ around U≈7, beyond which $E(\rho_{12})$ stabilizes to a constant value as *U* continues to increase.

For N=6 and U=0, the uneven distribution of electrons leads to increased entanglement $E(\rho_{ij})$ compared to N=4, as shown in Figure 8b. This is particularly evident for $E(\rho_{12})$, as sites 1 and 2 are more likely to adopt the |••,•〉 configuration instead of the local half-filled state. Figure 6i and Figure 7i illustrate that the electron arrangements |••,•,•,••〉, |••,◦,••,••〉, and |•,••,•,••〉 play a key role in determining the entanglement. For α=0.7, double occupancy is preferred, leading to a more rapid decline in configurations like |•,••,•,••〉 as *U* increases, which in turn causes a quicker reduction in entanglement $E(\rho_{ij})$ compared to α=0.2. As *U* enters the strong coupling regime, $E(\rho_{14})$ approaches zero for both the α=0.2 and α=0.7 cases, since sites 1 and 4 predominantly favor the |••〉 configuration. Similarly, $E(\rho_{12})$ and $E(\rho_{13})$ converge to constant values as sites 2 and 3 favor the |•〉 state. Notably, $E(\rho_{12})$ and $E(\rho_{13})$ remain larger than $E(\rho_{14})$ because sites 2 and 3 can occupy both |↑〉 and |↓〉 states, while, for sites 1 and 4, only one configuration becomes dominant as *U* increases, as shown in Figure 6i and Figure 7i.

### 4.3. Entanglement Analysis for $\varepsilon_{1}\neq 0$ with N=4

Altering the potential energy of a specific quantum dot can significantly impact the entanglement behavior in the system, as demonstrated in Figure 4, Figure 5, Figure 6 and Figure 7. For a particular quantum dot *i*, decreasing its potential energy causes electrons to congregate in this dot, which is reflected in the changes in the reduced density matrix elements: $v_{i,7}$, $v_{i,8}$, and $v_{i,9}$ increase, while $v_{i,1}$ to $v_{i,6}$ decrease.

In contrast, increasing the potential energy of dot *i* leads to the dispersal of electrons to other dots, resulting in a decrease in all matrix elements of $\rho_{i}$ except $v_{i,1}$, which corresponds to zero electron occupancy. In extreme cases, where the potential energy $\varepsilon_{i}$ undergoes significant changes, the electron configuration in this dot transitions to either |0〉 or ${|↑}_{g}{↓}_{g}{,↑}_{e}{↓}_{e}\rangle$, causing the local entanglement value to drop to zero, as shown in Figure 4a, Figure 5a, Figure 6a, and Figure 7a. This phenomenon is particularly pronounced in the weak coupling regime, where electrons have greater mobility.

Figure 4 shows the entanglement diagrams for four sites and four electrons under coupling strength ratio α=0.2, where the system favors the spin density wave at $\varepsilon_{1}=0$ [62,63,64,65]. Figure 4a depicts the relationship between the local entanglement $E(\rho_{1})$, potential energy $\varepsilon_{1}$, and interaction strength *U*. In the weakly coupled regime (U<1), as $\varepsilon_{1}$ deviates from zero, the value of $E(\rho_{1})$ rapidly decreases from approximately 2 to 0. Meanwhile, in the strongly coupled regime (U>30), electrons tend to remain separated in their respective quantum dots, adopting spin-wave-like configurations. Consequently, the local entanglement value approaches a limit of 1 as *U* increases. Here, we examine the system’s favorable occupancy configurations and their energies to understand its entanglement diagram behavior. In the regime where the potential energy $\varepsilon_{1}>0$, an increase in $\varepsilon_{1}$ at a constant *U* increases the system energy of the |•,•,•,•〉 configuration, which makes the lesser electron configuration in the first site prevail, inducing a transition in the main electron occupancy configuration components of the system’s ground states from mostly |•,•,•,•〉 to the collection of |◦,••,◦,••〉, |◦,••,•,•〉, and |◦,••,•,•〉. Consequently, in the weakly coupled regime (U<1), $E(\rho_{1})$ undergoes a rapid decline, exhibiting distinct boundaries, while $E(\rho_{2})$, $E(\rho_{3})$, and $E(\rho_{4})$ remain largely unchanged, as depicted in Figure 4a–d. It is noteworthy that, although the preferred electron occupancy configuration for dot 2 is |••〉, the influence of other occupancy configurations like |•〉 is also significant, as shown in Figure 4h, leading to a blurred boundary in $E(\rho_{2})$.

In the strongly coupled regime (U≫1 and $\varepsilon_{1}\ll U$), the system continues to favor the |•,•,•,•〉 occupancy configuration, where a substantial potential difference is required to alter the electron number configurations of the first site from one electron to another number. This transition is depicted in Figure 4a, where an orange belt precedes the red entropy area at $\varepsilon_{1}>0$. It results from a rapid shift in the preferred electron configurations, as shown in Figure 4h. Adjacent to this belt, three regimes can be distinguished based on the coupling strength and the extent of potential energy influence: (1) the potential energy-influenced weak coupling regime, where U∼1 and $\varepsilon_{1}∼U$, allows electrons to be easily influenced by the potential energy difference between dots; (2) the potential energy-influenced strong coupling regime, representing the transition between weak and strong coupling regimes, where the potential energy can readily shift the system’s favorable configurations; and (3) the strong coupling regime unaffected by the potential energy, where U≫1 and the system remains largely unchanged by the relatively minor potential energy differences.

In the regime where the potential energy $\varepsilon_{1}<0$, multiple entanglement belts exist, since one quantum dot can contain four electrons at most. In weakly coupled regimes, a decrease in potential energy $\varepsilon_{1}$ will quickly lead all electrons to be localized in site 1, since there are only four electrons in four quantum dots. More specifically, due to the size effect, the system is fully localized, and all entanglement values rapidly decline to zero, as shown in Figure 4a–g. As the coupling strength *U* increases, the system energy proportion from $\varepsilon_{1}$ decreases; therefore, as shown in Figure 4j, the favorable electron occupancy configurations of the ground states in the spin chain undergo a series of shifts: initially from $|\frac{••}{••},◦,◦,◦\rangle$ to $|\frac{•}{••},◦,•,◦\rangle$ and $|\frac{•}{••},◦,◦,•\rangle$ and then to |••,◦,•,•〉 and eventually to |•,•,•,•〉. Here, we use $\frac{•}{••}$ or $\frac{••}{••}$ to represent a site occupied by three electrons or four electrons, respectively.

Firstly, in the intermediate phase, where the preferred electron occupancy configurations are $|\frac{•}{••},◦,•,◦\rangle$ and $|\frac{•}{••},◦,◦,•\rangle$, three electrons tend to reside in the first dot, while the remaining electron occupies either the third or fourth dot. For these two configurations in our model, they have the same energy. Consequently, $E(\rho_{1})$, $E(\rho_{3})$, and $E(\rho_{4})$ exhibit higher local entanglement values, whereas $E(\rho_{2})$ declines to a lower value, as depicted in Figure 4a–d. This distribution demonstrates a transition in the preferred states across the quantum dots from 1 to 4. Specifically, the value of $E(\rho_{1})$ is associated with states indicative of three-electron occupancy $|\frac{•}{••}\rangle$, $E(\rho_{2})$ corresponds to zero electron occupancy |◦〉 (or |0〉), and $E(\rho_{3})$ and $E(\rho_{4})$ oscillate between one-electron occupancy |•〉 and zero occupancy |◦〉.

Secondly, when the system’s preferred occupancy configuration is |••,◦,•,•〉, the first dot favors double occupancy and the second dot favors zero occupancy, while the third and fourth dots favor one-electron occupancy. As a result, $E(\rho_{1})$, $E(\rho_{2})$, and $E(\rho_{12})$ approach zero, while $E(\rho_{3})$ and $E(\rho_{4})$ become similar, with high entanglement values as *U* increases.

Lastly, in the region where the system favors the |•,•,•,•〉 occupancy configuration, all entanglement behaviors align with those in the $\varepsilon_{1}\neq 0$ regime as the coupling strength becomes the dominant factor. Notably, the entanglement measures $E(\rho_{3})$ and $E(\rho_{4})$ exhibit smooth boundary transitions, indicating a preference for single-electron occupancy |•〉 in both the third and fourth quantum dots at this boundary. These regimes are more distinguishable in the pairwise entanglement $E(\rho_{ij})$, as depicted in Figure 4e–g. Near this belt (the potential energy-influenced strong coupling regime, $\varepsilon_{1}>0$), the states ${|0,↑}_{g}{↓}_{g}\rangle$ are highly favored for the pair $\rho_{12}$, resulting in a low entanglement value for $E(\rho_{12})$, while ${|0,↑}_{g}\rangle$ and ${|0,↓}_{g}\rangle$ are preferred for the pairs $\rho_{13}$ and $\rho_{14}$, leading to high entanglement values for $E(\rho_{13})$ and $E(\rho_{14})$. In the strongly coupled regime (U≫1 and $\varepsilon_{1}\ll U$), the system continues to favor the |•,•,•,•〉 occupancy configuration; therefore, similarly to the $\varepsilon_{1}=0$ case, the system shows a preference for the configurations ${|↑}_{g}{,↓}_{g}{,↑}_{g},{↓}_{g}\rangle$ and ${|↓}_{g}{,↑}_{g}{,↓}_{g},{↑}_{g}\rangle$ over other spin state configurations, resulting in $E\left(\rho_{12}\right)<E\left(\rho_{13}\right)∼E\left(\rho_{14}\right)$.

When the coupling strength ratio is set to α=0.7, the system exhibits a preference for double occupancy over single occupancy, where the system favors the charge density wave at $\varepsilon_{1}=0$ [62,63,64,65]. This preference is maintained even when $\varepsilon_{1}\neq 0$, as demonstrated in Figure 5h,j. For  $\varepsilon_{1}>0$, the favored electron occupancy configuration readily becomes |◦,••,◦,••〉 until $U\gg \varepsilon_{1}$, resulting in $E\left(\rho_{i}\right)∼E\left(\rho_{ij}\right)∼0$ (*i* for all sites from 1 to 4) when $U<\varepsilon_{1}$. Notably, in the weak coupling regime (U∼1), $E(\rho_{1})$ equals zero, while $E(\rho_{2})$, $E(\rho_{3})$, $E(\rho_{4})$, $E(\rho_{12})$, $E(\rho_{13})$, and $E(\rho_{14})$ experience a decrease in the entanglement value, caused by the reduction of the electron occupancy configuration |◦,••,•,•〉, as shown in Figure 5h.

For $\varepsilon_{1}<0$, the system similarly experiences three transitions, as illustrated in Figure 5j. With increasing *U*, the electron occupancy in site 1 changes from 4 to 2, resulting in variations in the entanglement values across all sites, as shown in Figure 5a–d. $E(\rho_{1})$ remains nonzero only when the average electron number in this dot is 3, due to the presence of two favored configurations, either up or down in the excited state. $E(\rho_{2})$ is predominantly zero as this site is typically unoccupied by electrons, except along the boundary line, where transitions between different system configurations render $E(\rho_{2})$ nonzero. Regarding $E(\rho_{3})$ and $E(\rho_{4})$, their electron configurations tend to converge in the strong coupling regime, resulting in similar entanglement behaviors. For $E(\rho_{12})$, the occupancy in site 1 influences the behavior of $E(\rho_{12})$, making it similar to $E(\rho_{1})$, as shown in Figure 5e. Figure 5f,g show, for $E(\rho_{13})$ and $E(\rho_{14})$, their behavior in the regime where $U\gg \varepsilon_{1}$ is similar to $E(\rho_{3})$ and $E(\rho_{4})$, respectively. When $U∼\varepsilon_{1}$, they also exhibit distinct features, similarly to $E(\rho_{1})$.

In conclusion, the system’s entanglement behavior, affected mainly by the coupling strength ratio, exhibits charge density wave and spin density wave configurations, where the system favors the spin density wave at $\varepsilon_{1}=0$ [62,63,64,65], and the energy variations from site 1 $\varepsilon_{1}$ and its ratio to the Coulomb interaction strength will also alter the system configuration when the system is based on the system energy.

### 4.4. Entanglement Analysis for $\varepsilon_{1}\neq 0$ with N=6

In contrast to the N=4 case, the N=6 system in a four-site lattice (L=4) inherently exhibits an imbalance in electron configurations, necessitating the consideration of additional configurations.

In the strong coupling regime, where $U\gg \varepsilon_{1}$, Figure 6h,j and Figure 7h,j demonstrate that, for both $\varepsilon_{1}>0$ and $\varepsilon_{1}<0$, and for coupling ratios α=0.2 and α=0.7, due to the extra electrons, the system’s favored occupancy configuration is |••,•,•,••〉, which has the minimum energy. This occupancy configuration leads to both $E(\rho_{1})$ and $E(\rho_{4})$ becoming zero, while $E(\rho_{2})$ and $E(\rho_{3})$ share the same entanglement value of approximately 1.2. When $U∼\varepsilon_{1}$ and $\varepsilon_{1}>0$, the most favorable occupancy configuration for both α=0.2 and α=0.7 is |•,••,•,••〉, leading to $E\left(\rho_{1}\right)∼E\left(\rho_{3}\right)$ and $E\left(\rho_{2}\right)∼E\left(\rho_{4}\right)$, as shown in Figure 6a–d and Figure 7a–d. For $\varepsilon_{1}<0$, the most favorable occupancy configuration is $|\frac{•}{••},•,•,•\rangle$ for α=0.2, and, for α=0.7, the configurations $|\frac{•}{••},◦,••,•\rangle$ and $|\frac{•}{••},◦,•,••\rangle$ are preferred. For α=0.2, the |•〉 configuration of site 2 leads $E(\rho_{2})$ and $E(\rho_{12})$ to become nonzero, which is the opposite for α=0.7, since site 2 favors the |◦〉 configuration. Therefore, $E(\rho_{2})$ becomes zero and $E(\rho_{12})$ behaves like $E(\rho_{1})$, as shown in Figure 6e.

In the weak coupling regime with $\varepsilon_{1}>0$, the system prefers specific electron configurations based on the coupling strength ratio α. For α=0.2, the favored configurations are |◦,••,••,••〉 and $|◦,••,•,\frac{•}{••}\rangle$, while, for α=0.7, the preferences shift to |◦,••,••,••〉 and $|◦,\frac{•}{••},◦,\frac{•}{••}\rangle$. As *U* increases within this regime, a transition occurs: for α=0.2, the system changes towards occupancy |◦,••,••,••〉, causing all entanglement measures $E(\rho_{i})$ and $E(\rho_{ij})$ to vanish. In contrast, for α=0.7, the system evolves towards the configuration $|◦,\frac{•}{••},◦,\frac{•}{••}\rangle$, leading to the vanishing of $E(\rho_{1})$, $E(\rho_{3})$, and $E(\rho_{13})$, while $E(\rho_{2})$, $E(\rho_{4})$, $E(\rho_{12})$, and $E(\rho_{14})$ stabilize at a constant value.

In the weak coupling regime with $\varepsilon_{1}<0$, all four electrons are in the first dot. Both for α=0.2 and α=0.7, the system exhibits a preference for the configurations $|\frac{••}{••},◦,•,•\rangle$ and $|\frac{••}{••},◦,••,◦\rangle$, respectively. As a result, in these two coupling ratio settings, the entanglement measures $E(\rho_{i})$ and $E(\rho_{ij})$ display similar patterns: $E(\rho_{1})$ remains at zero, and $E(\rho_{2})$ and $E(\rho_{12})$ gently descend to zero, while $E(\rho_{3})$, $E(\rho_{4})$, $E(\rho_{13})$, and $E(\rho_{14})$ find equilibrium at a constant value. Notably, the values of $E(\rho_{i})$ and $E(\rho_{ij})$ differ between α=0.2 and α=0.7, caused by the different electron configuration ratios.

### 4.5. Entanglement Comparison for Larger Systems

**Figure 9:**
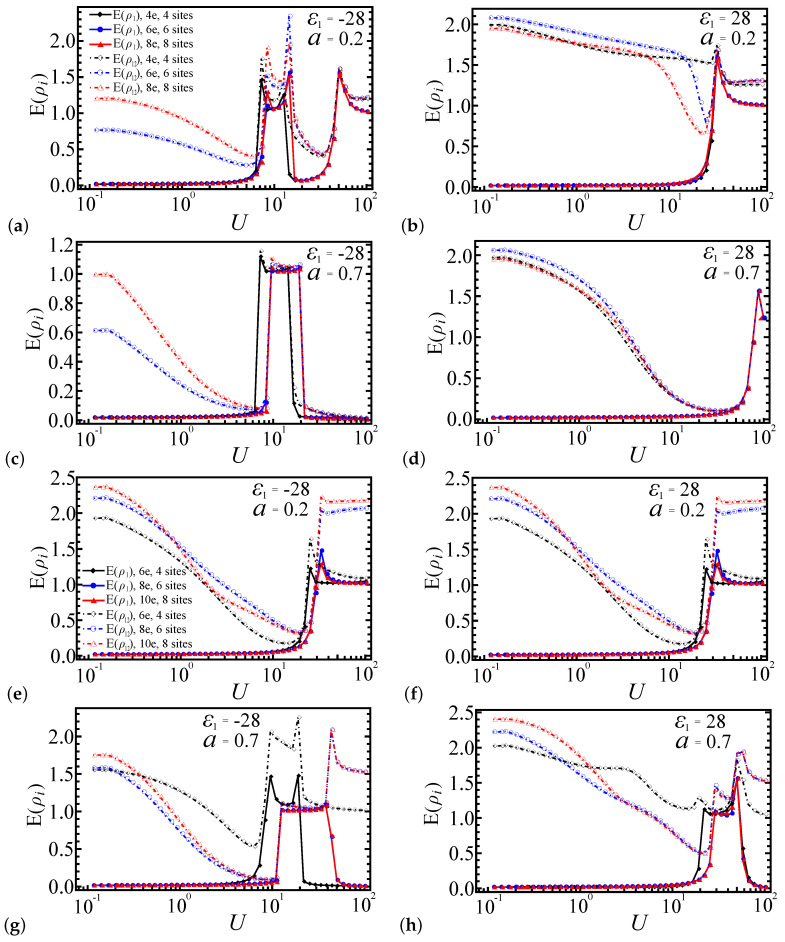
Entanglement profiles of $E(\rho_{1})$ and $E(\rho_{12})$ as a function of interaction strength *U* for L=4, L=6, and L=8 quantum dot spin chain systems. Solid lines represent $E(\rho_{1})$ and dashed dot lines represent $E(\rho_{12})$. (**a**,**b**) N=L, α=0.2; (**c**,**d**) N=L, α=0.7; (**e**,**f**) N=L+2, α=0.2; (**g**,**h**) N=L+2, α=0.7.

In this section, we extend our analysis by calculating the entanglement as a function of the interaction strength *U* for larger systems with L=6 and L=8, and we compare these results with the L=4 case. While the smallest system size L=4 provides initial insights into the entanglement behavior, it is essential to investigate larger systems to ensure that the observed phenomena are not merely artifacts of finite-size effects. We restrict our calculations to a system size of N=8 using the exact diagonalization method. For larger systems, the number of possible electron configurations involving multiple orbits increases exponentially with the system size, leading to substantial computational resource demands. Figure 9a–d correspond to systems with electron number N=L, while Figure 9e–h correspond to systems with N=L+2. For Figure 9b,d,f,h, the potential energy of the first dot is set to $\varepsilon_{1}=28$, whereas, for Figure 9a,c,e,g, it is set to $\varepsilon_{1}=-28$. The coupling strength ratio α is set to 0.2 in Figure 9a,b,e,f and to 0.7 in Figure 9c,d,g,h. In all figures, the entanglement profiles are color-coded as follows: black represents the four-site system (L=4), blue represents the six-site system (L=6), and red represents the eight-site system (L=8). The solid lines depict the local entanglement $E(\rho_{1})$, while the dot-dashed lines represent the pairwise entanglement $E(\rho_{12})$. Each curve contains 50 data points for comparison, denoted by different shapes.

Figure 9a,b illustrate the entanglement measures $E(\rho_{1})$ and $E(\rho_{12})$ as functions of *U* for $\varepsilon_{1}=28$ and $\varepsilon_{1}=-28$. For $\varepsilon_{1}=28$, both $E(\rho_{1})$ and $E(\rho_{12})$ exhibit a shift with increasing *U*, while, for $\varepsilon_{1}=-28$, they display three shifts, consistent with the prior results for L=4. In the strong coupling regime ($U\gg \varepsilon_{1}$), the entanglement values for larger systems (L=6 and L=8) converge to a stable curve, whereas the L=4 system shows qualitatively similar behavior but with less sharp transitions due to finite-size effects. As the system size increases from L=4 to L=8, the entanglement measures undergo sharper transitions, reflecting a reduction in finite-size effects and a closer approximation to the thermodynamic limit, where phase boundaries are more precisely defined. Similarly, Figure 9c,d reveal two and one entanglement shifts, respectively, for α=0.7, highlighting the role of the coupling strength ratio in the system’s favorable electron configurations. These abrupt shifts in entanglement align with the emergence of favored system configuration phases.

For systems with two extra electrons (N=L+2), as depicted in Figure 9e–h, the pairwise entanglement $E(\rho_{12})$ decreases more rapidly under a weak coupling ratio (α=0.2) compared to a strong coupling ratio (α=0.7). Specifically, for α=0.2, Figure 9e,f each reveal a single entanglement shift. In the strong coupling regime, the entanglement increases with larger system sizes *L*. For α=0.7, Figure 9g displays three sharp entanglement shifts, while Figure 9h shows smoother, more unstable shifts that become sharper as the system size increases. The local entanglement $E(\rho_{1})$ exhibits a similar pattern of abrupt shifts, with these shifts occurring earlier in smaller systems (L=4) due to finite-size effects and the presence of additional electrons. Similarly, in certain regimes, the entanglement values rise with an increasing system size (L=6 and L=8), and the transitions become more pronounced.

Due to the limitation of the system size, the entanglement behavior here differs from that of larger systems, caused by system-favorable configurations and the configuration energy. This effect can be seen in Figure 9: for L=6 and L=8, the entanglement behavior is nearly identical under different parameters, whereas, for the L=4 system, despite following similar qualitative trends, it displays quantitative differences, particularly in the entanglement peak positions.

### 4.6. Boundaries of Entanglement Diagrams for Large Systems

In this section, we expand the entanglement diagram from a small, finite-size system to a larger spin chain quantum dot system. It is evident from the ground state of the finite-size system that advantageous electron configurations significantly influence the boundaries and values of the entanglement diagram. This analysis can be readily extended to larger systems by calculating the energy of the electron configuration obtained from the Hubbard model (see Equation (1)). Since the system always favors the configuration with the lowest energy, which can be easily calculated and observed in small systems, we use this principle to infer the most favored configuration in larger systems.

**Figure 10:**
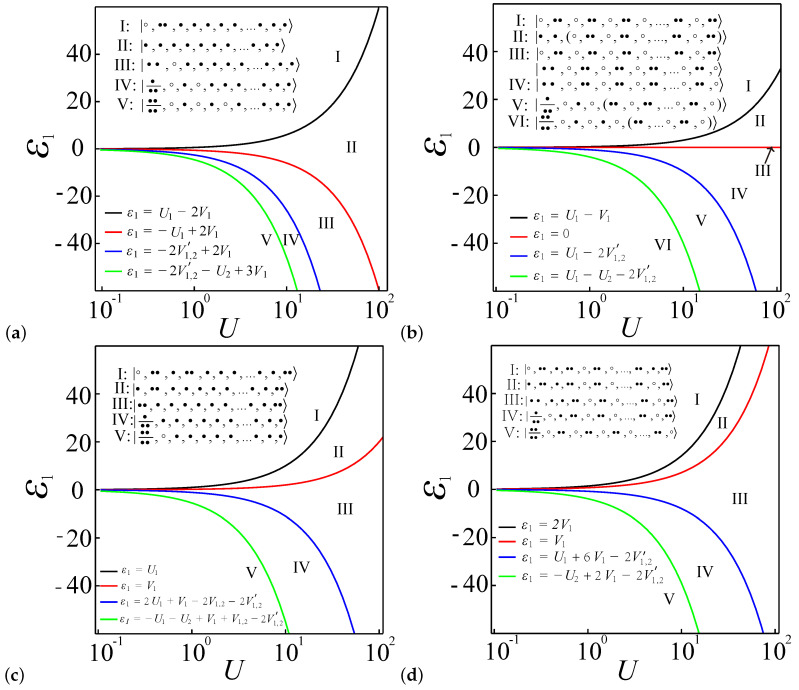
The boundaries in the entanglement diagrams for large systems, which are derived by analyzing the energy of dominant configurations as determined by the EHM. For N=L, (**a**) α=0.2, (**b**) α=0.7. For N=L+2, (**c**) α=0.2, (**d**) α=0.7.

For the case where α=0.2 and N=L, with *L* denoting the length of the spin chain and indicating an average of one electron per quantum dot, the system exhibits a preference for single occupancy at each quantum dot, resulting in a spin density wave structure, as described in previous studies [62,63,64,65]. Figure 10a depicts the evolution of the dominant system configurations as the potential energy $\varepsilon_{1}$ transitions from positive to negative values, showcasing a sequence of dominant configurations across regimes **I** to **V**:

(10a) $$\begin{array}{cc} I: & \;|◦,••,•,•,•,•,•,...•,•,•\rangle , \end{array}$$

(10b) $$\begin{array}{cc} II: & \;|•,•,•,•,•,•,•,...•,•,•\rangle , \end{array}$$

(10c) $$\begin{array}{cc} III: & \;|••,◦,•,•,•,•,•,...•,•,•\rangle , \end{array}$$

(10d) $$\begin{array}{cc} IV: & \;|\frac{•}{••},◦,•,◦,•,•,•,...•,•,•\rangle , \end{array}$$

(10e) $$\begin{array}{cc} V: & \;|\frac{••}{••},◦,•,◦,•,◦,•,...•,•,•\rangle . \end{array}$$

The energies associated with these configurations, as derived from the Hubbard model, are as follows: **I**: $U_{g}+\left(N-3\right)V_{g}$, **II**: $\left(N-1\right)V_{g}+\varepsilon_{1}$, **III**: $\left(N-3\right)V_{g}+U_{g}+2\varepsilon_{1}$, **IV**: $\left(N-5\right)V_{g}+U_{g}+2V_{g,e}^{\prime }+3\varepsilon_{1}$, **V**: $\left(N-7\right)V_{g}+U_{g}+U_{e}+4V_{g,e}^{\prime }+4\varepsilon_{1}$. Consequently, the boundaries distinguishing these regions in Figure 10a can be calculated as follows:

(11a) $$\begin{array}{cccc} I\text{-}II: & \; & \varepsilon_{1} & =U_{g}-2V_{g}, \end{array}$$

(11b) $$\begin{array}{cccc} II\text{-}III: & \; & \varepsilon_{1} & =-U_{g}+2V_{g}, \end{array}$$

(11c) $$\begin{array}{cccc} III\text{-}IV: & \; & \varepsilon_{1} & =-2V_{g,e}^{\prime }+2V_{g}, \end{array}$$

(11d) $$\begin{array}{cccc} IV\text{-}V: & \; & \varepsilon_{1} & =-2V_{g,e}^{\prime }-U_{e}+3V_{g}. \end{array}$$

The boundaries between different regions mark the transitions between different electron occupancy configurations. In region **I**, the system exhibits a preference for the configuration |◦,••,•,•,•,•,•,...•,•,•〉. This indicates that the first dot is unoccupied when $\varepsilon_{1}>U_{g}-2V_{g}$, and the extra electron from the first dot is likely to be found either in the second dot or at the last dot of the spin chain. This preference arises because the electron at these positions contributes only one $V_{\nu }$ interaction, while electrons in other positions contribute to $2V_{\nu }$ interactions. Similarly, in regions **III**, **IV**, and **V**, when the first dot accommodates more than one electron, the second dot tends to be unoccupied. This arrangement minimizes the system energy generated from Coulomb interactions between the first and second dots. Similarly, for the remaining dots, electrons tend to favor configurations where both neighboring dots are unoccupied and form spin density wave configurations, reducing the overall Coulomb interaction terms within the system.

Second, for α=0.7 with N=L, the system adopts a charge density wave structure [62,63,64,65]. Figure 10b illustrates the progression of the dominant system configurations as the potential energy $\varepsilon_{1}$ shifts from positive to negative values, depicting a sequence of configurations that emerge in this transition. The configurations are as follows:

(12a) $$\begin{array}{cc} I: & \;|◦,••,◦,••,◦,••,◦,...,••,◦,••\rangle , \end{array}$$

(12b) $$\begin{array}{cc} II: & \;|•,•,(◦,••,◦,••,◦,...,••,◦,••)\rangle , \end{array}$$

(12c) $$\begin{array}{cc} III: & \;|◦,••,◦,••,◦,••,◦,...,••,◦,••\rangle , \end{array}$$

(12d) $$\begin{array}{cc} & \;|••,◦,••,◦,••,◦,••,...◦,••,◦\rangle , \end{array}$$

(12e) $$\begin{array}{cc} IV: & \;|(••,◦,••,◦,••,◦,••,...◦,••,◦)\rangle , \end{array}$$

(12f) $$\begin{array}{ccc} V & : & \;|\frac{•}{••},◦,•,◦,\left(••,◦,••,...◦,••,◦\right)\rangle , \end{array}$$

(12g) $$\begin{array}{ccc} VI & : & \;|\frac{••}{••},◦,•,◦,•,◦,\left(••,...◦,••,◦\right)\rangle . \end{array}$$

The energies corresponding to these configurations, as calculated from the Hubbard model, are as follows: **I**: $NU_{g}/2$, **II**: $\left(N-2\right)U_{g}/2+V_{g}+\varepsilon_{1}$, **III**: $NU_{g}/2$, **IV**: $NU_{g}/2+2\varepsilon_{1}$, **V**: $\left(N-4\right)U_{g}/2+U_{g}+V_{g,e}^{\prime }+3\varepsilon_{1}$ **VI**: $\left(N-6\right)U_{g}/2+U_{g}+U_{e}+4V_{g,e}^{\prime }+4\varepsilon_{1}$. Therefore, we can calculate the boundary functions of these regions in Figure 10b as

(13a) $$\begin{array}{cccc} I\text{-}II: & & \varepsilon_{1} & =U_{g}-V_{g}, \end{array}$$

(13b) $$\begin{array}{cccc} II\text{-}III: & & \varepsilon_{1} & =0, \end{array}$$

(13c) $$\begin{array}{cccc} III\text{-}IV: & & \varepsilon_{1} & =0, \end{array}$$

(13d) $$\begin{array}{cccc} IV\text{-}V: & & \varepsilon_{1} & =U_{g}-2V_{g,e}^{\prime }, \end{array}$$

(13e) $$\begin{array}{cccc} V\text{-}VI: & & \varepsilon_{1} & =U_{g}-U_{e}-2V_{g,e}^{\prime }. \end{array}$$

In regions **I** and **IV**, the system adopts a global charge density wave structure, with the first dot being unoccupied and doubly occupied, respectively. Notably, in region **III**, the system exhibits a charge density wave pattern that arises from the superposition of two distinct configurations |◦,••,◦,••,◦,••,◦,...,••,◦,••〉 and |••,◦,••,◦,••,◦,••,...◦,••,◦〉. In region **II**, both the first and second dots host a single electron. This arrangement minimizes the Coulomb interaction terms compared to alternative configurations. Specifically, the potential energy shift in the first dot and its interaction with the second dot yield a lower energy of $\varepsilon_{1}+V_{g}$. In contrast, hosting two electrons in the second dot would result in higher energy, given by $U_{g}$, thus making the single-electron configuration energetically favorable. For regions **V** and **VI**, apart from the first dot, the system prefers configurations where neighboring dots are unoccupied, maintaining the charge density wave structure throughout the rest of the system.

Third, in the case of α=0.2 with N=L+2, the presence of two additional electrons raises the average electron count per dot above one. Consequently, only a portion of the system continues to exhibit a spin density wave structure. As illustrated in Figure 10c, the dominant system configurations evolve as the potential energy $\varepsilon_{1}$ transitions from positive to negative values. The sequence of dominant configurations for regions **I** to **V** is as follows:

(14a) $$\begin{array}{cc} I: & \;|◦,••,•,••,•,•,•,...•,•,••\rangle , \end{array}$$

(14b) $$\begin{array}{cc} II: & \;|•,••,•,•,•,•,•,...•,•,••\rangle , \end{array}$$

(14c) $$\begin{array}{cc} III: & \;|••,•,•,•,•,•,•,...•,•,••\rangle , \end{array}$$

(14d) $$\begin{array}{cc} IV: & \;|\frac{•}{••},•,•,•,•,•,•,...•,•,•\rangle , \end{array}$$

(14e) $$\begin{array}{cc} V: & \;|\frac{••}{••},◦,•,•,•,•,•,...•,•,•\rangle . \end{array}$$

The energy associated with each configuration in the Hubbard model is obtained as follows: **I**: $3U_{g}+\left(N-6\right)V_{g}+8V_{g}$, **II**: $\varepsilon_{1}+2U_{g}+\left(N-4\right)V_{g}+6V_{g}$, **III**: $2\varepsilon_{1}+\left(N-3\right)V_{g}+2U_{g}+4V_{g}$, **IV**: $3\varepsilon_{1}+\left(N-2\right)V_{g}+2V_{g}+V_{g,e}+2V_{g,e}^{\prime }$, **V**: $4\varepsilon_{1}+\left(N-3\right)V_{g}+U_{g}+U_{e}+4V_{g,e}^{\prime }$. Accordingly, the boundary functions distinguishing these regions in Figure 10c are calculated as

(15a) $$\begin{array}{cccc} I\text{-}II: & \; & \varepsilon_{1} & =U_{g}, \end{array}$$

(15b) $$\begin{array}{cccc} II\text{-}III: & \; & \varepsilon_{1} & =V_{g}, \end{array}$$

(15c) $$\begin{array}{cccc} III\text{-}IV: & \; & \varepsilon_{1} & =V_{g}+2U_{g}-V_{g,e}-2V_{g,e}^{\prime }, \end{array}$$

(15d) $$\begin{array}{cccc} IV\text{-}V: & \; & \varepsilon_{1} & =V_{g}+V_{g,e}-U_{g}-U_{e}-2V_{g,e}^{\prime }. \end{array}$$

In region **I**, the first dot is unoccupied, prompting the three additional electrons to distribute themselves along the chain to minimize Coulomb interactions: two electrons position themselves at the ends, while the third occupies a central position. This arrangement ensures minimal interaction with the electrons at the ends. Similarly, in regions **II** and **III**, the additional electrons also preferentially reside at the chain ends. Conversely, in regions **IV** and **V**, the extra electrons occupy the first dot, freeing up space along the rest of the chain for one electron per dot. Notably, in region **IV**, the electron in the second quantum dot remains localized rather than migrating to the third dot or further along the chain. This localization is evident when considering the configuration $|\frac{•}{••},•,•,•,•,•,•,\ldots ,•,•,•\rangle$, where the electron in the second dot interacts with its adjacent electron with energy of $3V_{g}+V_{g,e}$. Conversely, in the competitive configuration $|\frac{•}{••},◦,••,•,•,•,•,\ldots ,•,•,•\rangle$, the electron in the third dot interacts with the fourth dot with energy of $U_{g}+2V_{g}$. This results in higher total energy than the former configuration under the parameter setting α=0.2. In conclusion, as the potential of the first site increases, and more electrons move from the first dot to the neighboring dot, for $\varepsilon_{1}<0$, the remaining dot maintains the spin density wave structure. Meanwhile, for $\varepsilon_{1}\geq 0$, due to the extra two electrons, the system maintains the spin density wave structure, except at the end sites.

Lastly, in the case of α=0.7 with N=L+2, shown in Figure 10d, regarding the dominating system configuration as $\varepsilon_{1}$ changes from $\varepsilon_{1}>0$ to $\varepsilon_{1}<0$, the configurations will appear as the following sequences:

(16a) $$\begin{array}{cc} I: & \;|◦,••,•,••,◦,••,◦,...,••,•,••\rangle , \end{array}$$

(16b) $$\begin{array}{cc} II: & \;|•,••,•,••,◦,••,◦,...,••,◦,••\rangle , \end{array}$$

(16c) $$\begin{array}{cc} III: & \;|••,•,•,••,◦,••,◦,...,••,◦,••\rangle , \end{array}$$

(16d) $$\begin{array}{cc} IV: & \;|\frac{•}{••},◦,•,••,◦,••,◦,...,••,◦,••\rangle , \end{array}$$

(16e) $$\begin{array}{cc} V: & \;|\frac{••}{••},◦,••,◦,••,◦,••,◦,...,••,◦\rangle . \end{array}$$

The energies corresponding to these configurations, as calculated from the Hubbard model, are as follows: **I**: $NU_{g}/2+8V_{g}$, **II**: $\varepsilon_{1}+NU_{g}/2+6V_{g}$, **III**: $NU_{g}/2+U_{g}+8V_{g}+2\varepsilon_{1}+NU_{g}/2+V_{g}+4V_{g}+2\varepsilon_{1}$, **IV**: $3\varepsilon_{1}+NU_{g}/2+2V_{g}+2V_{g,e}^{\prime }$, **V**: $4\varepsilon_{1}+NU_{g}/2+U_{e}+4V_{g,e}^{\prime }$. Consequently, the boundaries distinguishing these regions in Figure 10d can be calculated as follows:

(17a) $$\begin{array}{cccc} I\text{-}II: & \; & \varepsilon_{1} & =2V_{g}, \end{array}$$

(17b) $$\begin{array}{cccc} II\text{-}III: & \; & \varepsilon_{1} & =V_{g}, \end{array}$$

(17c) $$\begin{array}{cccc} III\text{-}IV: & \; & \varepsilon_{1} & =3V_{g}-2V_{g,e}^{\prime }, \end{array}$$

(17d) $$\begin{array}{cccc} IV\text{-}V: & \; & \varepsilon_{1} & =2V_{g}-U_{e}-2V_{g,e}^{\prime }. \end{array}$$

In regions **I** and **II**, the additional electrons—two in the former and one in the latter—have the flexibility to occupy any available sites along the spin chain. In region **III**, a distinctive arrangement emerges where two electrons specifically occupy sites 2 and 3. This localized occupation maintains a charge density wave structure throughout the remainder of the spin chain. The region **IV** exhibits a situation in which a single electron favors site 3, which is advantageous as it minimizes the Coulomb interaction, involving only a $2V_{\nu }$ contribution from the adjacent site 4, thereby optimizing the energy configuration. Finally, the region **V** naturally evolves into a global charge density wave structure, where the electron distribution systematically alternates along the entire chain, reflecting a stable and energetically favorable arrangement. In conclusion, as the potential of the first site increases, for region **II** to region **V**, the remaining dot maintains the charge density wave structure, except at the left end sites. Meanwhile, for region **I**, due to the extra two electrons, the system maintains the spin density wave structure, except at the first to third sites and the last two sites.

## 5. Conclusions

In this study, we systematically explored the entanglement properties of semiconductor quantum dots within a multi-site lattice, described by the EHM. Our investigations demonstrate that local and pairwise entanglement measures respond sensitively to interactions between Coulomb forces and tunneling effects, which are influenced by the system’s electronic configurations and variations in external potential energies. Notably, the entanglement characteristics show distinct transitions, influenced heavily by the coupling strength ratios and variations in the potential energy. We observed that varying the potential energy of a specific dot decisively altered the ground state configurations and, consequently, the entanglement measures, a phenomenon that is pronounced in both weak and strong coupling regimes. Extending our analysis to larger systems with L=6 and L=8, we found that these trends persisted, with sharper entanglement transitions emerging as the system size increased, suggesting reduced finite-size effects and enhanced control over the entanglement properties. This indicates that potential energy modifications can effectively control entanglement in quantum dot systems across various scales, which could be measured with indirect methods like machine learning [68,69,70,71]. Furthermore, tunable entanglement has potential applications in quantum state transfer protocols and spin shuttling in quantum dot arrays [75,76,77,78].

## Data Availability

The data presented in this study are available on request from the corresponding author.
